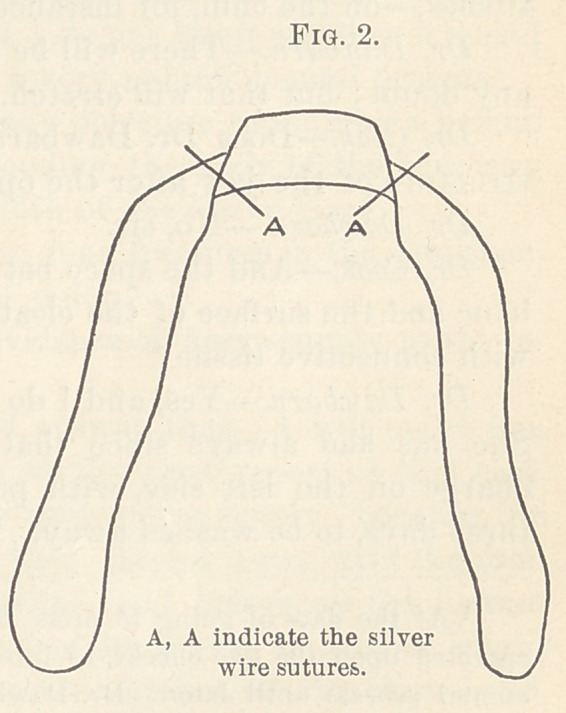# The New York Institute of Stomatology

**Published:** 1897-10

**Authors:** 

**Affiliations:** The New York Institute of Stomatology


### THE NEW YORK INSTITUTE OF STOMATOLOGY.

A REGULAR meeting of the Institute was held Tuesday evening, June 1, 1897, at the residence of Dr. A. H. Brockway, No. 13 Greene Avenue, Brooklyn, New York. The President, Dr. George Allan, occupied the chair.

The minutes of the last meeting were read and approved.

COMMUNICATIONS ON THEORY AND PRACTICE.

*The President.*—I understand that Dr. Geran has consented to report upon an interesting case he has recently been connected with, and we will be pleased to hear from him.

*Dr. J. P. Geran.*—It affords me much pleasure to meet with the Institute this evening, and I appreciate the privilege of making a few crude remarks relative to a case of fracture that fell into my hands a few months ago.

The patient under consideration is the daughter of Mr. B., of Brooklyn. She is about ten years of age, and one of the prettiest little girls I have ever seen.

The accident occurred December 9, 1896, in the following manner: Leaving her home after luncheon for school, looking diagonally across the street she espied a playmate, and, eager to meet her chum, she started to run at a rapid rate. Nearing the opposite side of the street she fell, striking the curb-stone, lacerating the left side of the upper lip fully three-fourths of an inch, vertically, in two places, cutting through to the labial surface of the alveolus, crushing the bone, and destroying the gum covering both superior centrals and the left lateral. The right central was knocked out and afterwards found on the sidewalk. The left central and lateral, with a portion of the palatal surface of the bone and soft tissue, were driven backward and hung by the gum covering the roof of the mouth.

Dr. S. H. Benton, of Bergen Street, a skilful physician and surgeon, was called in, and, judging that dental assistance would be needed, he sent for me. The child was given an anæsthetic, and, after carefully setting the fractured bone and teeth in their proper places, I took an impression of all the teeth in the upper jaw, using a quick-setting compound. I did not think it advisable to use plaster of Paris for the impression, on account of the liability of the particles to get into the throat; and, again, as I really desired an impression of only about half of the labial surface of the four incisors, or just enough to hold them in, I concluded the compound was the better to use. Had the labial surface been covered, as well as the buccal surface of the posterior teeth, it would have been impossible to know positively that the teeth were properly in place within the splint. To be doubly sure of this, I made a small opening in the splint at the cutting-edges. After restoring the teeth to their sockets and occluding the inferior and superior molars, I found a considerable overlapping of the inferior incisors by the superior, which enabled me to make the opening in the splint and do without a double plate.

The tendency in these cases, especially with children, after the bandage is on, and after becoming comparatively comfortable, is to make an effort to open the mouth, and if this can be done, which will readily be seen, the bandages can be tightened.

By making the plate with very little material between the molars and openings over the incisors, it enabled me to be sure of a good antagonism after removing the fixture.

After getting the model in shape, I followed the course that some text-books recommend,—that is, to burnish over the teeth two pieces of tin-foil, so as to enlarge the teeth, thereby allowing the splint to slip over them easily. This I found to be a mistake.

When I put the fixture in place I found it too loose, and had to warm the edges slightly and bend them in so as to bind on the molars in order to keep it in place, especially while the patient was under the anæsthetic and until we could get the bandage on. The splint was not allowed to extend over the molars beyond the free margin of the gum.

As both the superior and inferior deciduous cuspids and molars were lost in the accident, it allowed me to curve out the plate sufficiently to have a space to administer nourishment and disinfectants. This is another reason why I could make the splint thin, or, in other words, have very little vulcanite between the masticating surfaces.

Concerning the surgeon who had the case in charge, I will say that in a matter requiring so much delicate manipulation I would not wish to be associated with a more competent and gentle man than Dr. Benton has proved himself to be. While it was the occasion of our first meeting, the experience has caused me to be thoroughly satisfied that if there were a more friendly and fraternal feeling existing between men of the two professions, it would be to the advantage of both, and more especially to our patients.

I have asked Dr. Benton to add a few notes on this case, and he kindly sends me the following:

"Dr. Geran has asked me to add a postscript to his paper to be read before your society to-night. I take it that there is very little to add that the doctor has not already touched upon, and I know how prosy it would be for all to listen to a repetition on the same subject.

"Dr. Geran will doubtless give a history of the anatomical lesion from the stand-point of the dentist, and my notes will be principally surgical.

"When I first saw the child she presented a frightful appearance. After cleansing her face I found an extensive laceration of the labial tissue in all directions, which did not promise well for her future appearance.

"On examining the mouth I found what appeared to be the whole of the left upper jaw driven back almost to the palatal arch and broken into several pieces, leaving the roof of the mouth almost denuded, with one or two teeth lost. I realized at once that I must have a splint made by a dentist if I hoped to get anything like a satisfactory result.

"Dr. Geran kindly came to my relief. After the splint was made, which required about eight hours to get satisfactory, I adjusted the fragments, which we found difficult to keep in place, and Dr. Geran deftly adjusted the splint; I immediately repaired the soft parts. The whole time for dressing this injury extended from 1 to 11 P.M. One of the teeth was out all this time and returned to its place at the final dressing by Dr. Geran.

"The gum and bone over the front teeth were cut off or lost in some way, but at this writing are almost fully restored. The teeth seem to be solid, are even, and in every way as satisfactory as before. To keep the splint in its proper position I improvised, after many trials, a head-piece attached to a splint on the inferior maxillary, making even pressure along the whole course of the jaw. This was kept very tight, so there was no movement of the jaw whatever. The after-treatment was on general principles of cleanliness.

"I could not hope for the results which were attained without just the measures that were used. This case has seemed to create some interest in dental circles, and I trust Dr. Geran will be accorded the credit for the satisfactory results.

"I am glad that we have men in the dental profession who can go out of the usual routine of dental work and be of such great assistance to the surgeon.

(Signed) "S. H. BENTON, M.D."

*Dr. Geran.*—I tried this evening to procure a lower maxillary splint to show here, like the one used in this case, to hold the jaws firmly together; but as they are not kept in stock I was unable to do so. The one used for this case was made to order, and was so arranged as to do away with the old method of covering the entire head and part of the face with three or four yards of muslin bandage.

*Dr. S. E. Davenport.*—Would Dr. Geran be willing to give us a description of the teeth of the little girl as they were when he last saw her?

*Dr. J. P. Geran.*—I saw her yesterday, and the restoration or deposition of new bone and gum covering the labial surface of the teeth was remarkable.

I would like to state that, in my hurry when I was called to her residence to see the condition of things, I forgot to put the tooth which was knocked out in my pocket before I returned home to get my impression materials. Had I done so I would have removed the pulp, but it did not occur to me until after I returned to take the impressions. However, I thought I would put it in and trust to results. As already stated, the teeth grew fast, nearly covered with the bone and gum; but a few days ago the patient came to my office, and upon examining her mouth I found a fistula over the right central,—the tooth that had been knocked out.

I made an appointment with her for yesterday. She is a very timid little girl, and I asked the father to come with her and I would remove the pulp. I did so.

The left central and lateral that were dislocated and which hung by the gum, also the right central that was entirely out, are in an apparently healthy condition,—no discoloration whatever.

*Dr. C. A. Woodward.*—I have a little object-lesson which I wish to present that may be of service to young men, or to careless operators, as I presume there are some in existence. It is a case of two superior bicuspids that had porcelain crowns put upon them. The root of the one I hold here, as will be seen, is quite large and perfectly straight, but still the canal made for a Bonwill crown was drilled through the root, and the screw which was inserted passed through that opening into the soft tissue, and was in that position for three years, to the great discomfort of the patient. She told me there has been scarcely a month during the three years that she has not been to the dentist, who put on the crowns, for the treatment of those teeth. When I examined the case I found that one root had been perforated, and I made up my mind to extract both roots. The whole plate of the alveolar process was found to be destroyed, and still it had been treated for three years, and the dentist had hopes of curing it, which I cannot understand. In selecting teeth for implantation I chose as long roots as I could obtain, but not being able to get much of a hold in the process, as so much of it had been destroyed, I am in doubt about their success, although the operation was performed about six weeks ago, and the teeth are pretty firm. She says she has been able to masticate upon them, but I warned her about using them very much at present, as the hold is not sufficient. This work was not done in a so-called "dental parlor," but by a man who for years has prided himself upon being a very rapid operator.

*Dr. J. W. Russell.*—I have here the cast of a case which came into one of our hospitals this spring. She was a young lady, about twenty-two years old, who had had a great deal of trouble on one side of her face for about seven years. There was a lump there, and it kept growing larger and larger. I made a diagnosis of dentigerous cyst. Not being on the staff, I could not operate on the case, but I was present at the operation. There was nothing found in the cyst. It ran forward almost under the central incisor. The cavity was about three-quarters of an inch deep. It was opened, and the parts pressed together and packed, and it is now almost well, after about three months.

*Dr. F. Milton Smith.*—I have some very thin finishing strips that have been useful to me, and I have brought them along as samples. I suppose many of the gentlemen have used them; they are made of architect's tracing-cloth. In finishing amalgam fillings, before the amalgam is set, they are extremely convenient. I have also found them convenient for polishing. I get a yard or two of the architect's tracing-cloth, which may be obtained at most stationers', and get my printer to cut it for me. I have never come across anything as thin as this material with as much strength.

*The President.*—May we now have the pleasure of hearing from Dr. Dawbarn?

*Dr. R. H. M. Dawbarn.*—This is a rather premature report, for which I must apologize, but it is made at the request of Dr. Bogue. I hope to make a fuller one later on, accompanied with photographs of the abnormal condition, together with casts of the jaws and photographs of what results I may succeed in obtaining. The patient is a child of thirteen, daughter of a prominent Spanish family in Brooklyn, a patient of Dr. Hanning, and was referred to me for the surgery in the matter by the courtesy of Dr. Parker and Dr. Gage. There are two absolutely distinct conditions, and at least three operations of a surgical nature are necessary. The first is for enormous tonsils, so large that they nearly meet in the median line, and the pharyngeal lymphoids almost fill the pharynx. A doctor here in town has treated the child for some time with douches for catarrh, and did not pay attention to the fact that it was caused by this trouble, which between five and ten per cent. of children have in this climate. It is a common cause of high arching of the palate. The child is a mouth-breather, and, like most mouth-breathers, anæmic. I have removed the tonsils and cleaned out the pharynx thoroughly, and as soon as she is well I will treat her for the trouble about which I want to speak this evening. When she was about three or four years of age she had a severe attack of scarlet fever. I cannot get the exact details, because her present family doctor is not the one they had at that time. In consequence of that attack of scarlet fever severe necrosis of the lower jaw on both sides occurred, resulting in a sequestrum, which was cast off on both sides, consisting of the entire body of the lower maxilla, with the exception of the genial process and the two rami. The result shows, however, that the periosteum could not have been destroyed, because bone has been reproduced. There is a solid jaw, the normal ramus, and the normal genial process, and new bone formed from the periosteum which was left; but the periosteal cavity must have fallen in as far towards the tongue as the tongue would permit, as there is a distinct hollowing on both sides. All the teeth were lost, except in the region which was not attacked, which contains a set of seven teeth,—three incisors, two canines, and two bicuspids. Because of the other trouble, the narrow, high arch of the jaw, the upper front teeth project, and they project even more noticeably than they otherwise would because the chin recedes. Not only did the lower jaw fall in on each side, but, there being no support to the chin, the chin fell backward, so that the child's upper incisors protrude two-thirds of an inch beyond the incisors of the lower jaw. She has rather fat cheeks, and that makes still more striking the deformity on each side, because the scars in the regions from which the bone was removed have grown to the bone, making a deep depression on each side. The result is hideous in a child who would otherwise be beautiful, having large, dark eyes and expressive features.

Now, the problem that presents itself is what to do for the deformity on either side of the soft parts, and what to do for the lower jaw. I will tell what I contemplate doing, and then ask for suggestions. I propose to dissect out the scar and free it from the bone; but, with the tendency of scarred tissue to contract, I fear it will sink down again, and I contemplate at the time of the operation, before I absolutely finish the stitching, to inject into the cavity beneath (there will be a space of nearly an inch) freshly melted and sterilized vaseline as a partial support for these soft parts, and to prevent the scar reuniting to the bone. I have not done that in a similar case, but it is a standard treatment among surgeons at this day in certain cases of suppurating bubo. It is customary to squeeze gently or scrape out the contents through an incision only one-fifth of an inch long, and then fill the cavity with melted vaseline and five per-cent. iodoform, and put on a compress. The vaseline and iodoform mixture is absorbed and its place taken by connective tissue. We know, however, that the pus in buboes is not infrequently sterile,—that is, different from other pus in not containing microbes. When the child has recovered from those two operations, the question will arise whether we can do anything for the sinking in of the body of the jaw and the recession of the mental (genial) process,—whether I can bring that chin in some sense forward so that the teeth will more nearly articulate with the other jaw, and whether I cannot widen the lower jaw.

Fig. 1.

The arrow shows the direction in which the mental process is to be drawn.

Fig. 2.

A, A indicate the silver wire sutures.

This sketch indicates roughly the shape of the body and mental process of the lower jaw seen from above, the sinking in on either side being quite noticeable. I have indicated roughly what would be the position of the teeth, and what I contemplate doing is, with a slender saw, sawing along these black lines obliquely so my saw-cuts will be wider apart at the back than at the front, and, following the direction of this arrow, driving that detached mental portion forward and then suturing with silver wire. I will bring the chin more prominently forward in that way, and, because the bony wedge thus formed is wider at the back than at the front, I will inevitably have separated the two sides to a noticeable extent. By suturing that as aseptically as I can with silver wire, I ought to get union, the same as in a simple fracture of the jaw. I would like the opinion and suggestions of the gentlemen present as to that technique.[1]

*Dr. Houghton.*—Is the contour of the upper lip normal?

*Dr. Dawbarn.*—Practically so; the mother has kindly consented that the photographs that I have taken, and those to be taken later on, may be shown to this society.

*The President.*—Does Dr. Dawbarn know the relation existing between the lower jaw and the upper jaw as to width?

*Dr. Dawbarn.*—The lower jaw is distinctly narrower, just how much I cannot state exactly,—maybe one-third of an inch on each side.

*The President.*—What effect will that operation have on the soft tissues,—on the chin, for instance?

*Dr. Dawbarn.*—There will be some little tension at first, without any doubt; but that will stretch.

*Dr. Cook.*—Does Dr. Dawbarn expect any change in the bony structure of the jaw after the operation is performed?

*Dr. Dawbarn.*—No, sir.

*Dr. Cook.*—And the space between the present condition of the bone and the surface of the cicatrix, after it is healed, will be filled with connective tissue?

*Dr. Dawbarn.*—Yes, and I do not think there will be any scar. She has had always since that time of scarlet fever a little discharge on the left side, with perhaps a drop of pus every two or three days, to be washed away. That, on being probed, leads to the

---

[1] At the date of going to press Dr. Dawbarn reported that he had now operated upon the two cheeks, as intended, reserving the operation upon the mental process until later. Dr. Dawbarn states that the operation upon the left cheek showed that he was wrong in thinking that the continual mucopurulent discharge from that point indicated a remaining piece of sequestrum. Upon chiselling into the body of the bone he discovered a large, perfectly developed, first molar tooth, lying in a little chamber entirely surrounded by dense bone. The tooth was so placed that its crown was directed inward towards the tongue, and its root outward towards the cheek. From the membranous sac surrounding it and lining this little chamber came the discharge, at one delicate point, of a cloaca, which had continued all these years. It is evident, from the presence of this tooth, that at least a little of the natural bone on this side, containing this tooth-germ, did not die, and came away with the rest of the sequestrum.

interior of the new bone. Evidently there must have been an old sequestrum, which has never been removed, having become surrounded and held by the involucrum. The latest theory is that it is the carbonic acid of the blood which has the power to dissolve dead bone and thereby separate dead bone from living bone. I shall have to chisel in and remove the dead bone and sterilize the cavity with peroxide of hydrogen before suturing the soft parts.

*The President.*—Will Dr. Dawbarn kindly sketch what would be the outline of the other jaw in relation to this?

*Dr. Dawbarn.*—Yes, sir; something as I now indicate.

*The President.*—Is it Dr. Dawbarn's intention to spring out these two parts in the direction shown?

*Dr. Dawbarn.*—Yes; when the central portion is brought forward the sides will separate in the directions I have indicated. As to drills, for the wire suturing, the best bone-drill is an ordinary ten-cent awl for most bone-work,—not dental. I could go in between the central and lateral incisors and cut more obliquely and get greater lateral width; but I do not know whether it would be wise to do it. It would make a very narrow mental process.

*Dr. Russell.*—Would there be any objection to making a partial fracture at each side, too, and bending the body of the bone outward there, thus adding to the width of the lower jaw?

*Dr. Dawbarn.*—There would be four fractures in the jaw then, and I would be afraid of a lack of union.

*Dr. Brewster.*—Is there any evidence of nerve-supply to the inferior teeth at the present time?

*Dr. Dawbarn.*—I wish I could answer that. I will make it a point to inquire, so I shall know at the next report of this case. It would be strange if there were such nerve-supply, because the inferior dental canal must have been carried away with the dead bone. It is a very interesting point, and brings up the further point whether this bone, having had its nerves destroyed, might heal with difficulty after operation, or whether, on the other hand, it may have gotten a new trophic nerve-supply from other sources.

*Dr. Howe.*—As this very interesting case involves necrosis, I would like to ask a question,—namely, Whether necrosis ever exists without separation of the dead from the living, thereby causing sequestra, and whether it ever takes place without suppuration? The question has a bearing upon the common statement that the borders of the alveolar processes are necrosed when pyorrhœa alveolaris exists. A case in which I was consulted recently, involving an opening into the antrum, also had that question as a factor in the diagnosis and treatment. There was an opening into the antrum from previous suppuration, at the apex of the root of a second bicuspid tooth. The root had been removed and the opening through the socket of that tooth into the antrum was the full size of the root of the tooth. There was a clear opening into the antrum, and yet a surgeon proposed to make another opening, higher up, towards the malar bone, on the ground of necrosis, which would require the cutting away of dead tissue. There was, at the time, no suppuration, and I did not acquiesce in the opinion that necrosis existed. There has seemed to me to be sometimes confusion with regard to the signs of necrosis, and I think Dr. Dawbarn will help us to a clearer understanding of what necrosis means.

*Dr. Dawbarn.*—I have never heard or read of a case of necrosis in general surgery without suppuration and without ultimate reparation of bone. The usual period at which a surgeon expects that dead bone will be loosened is three months. Sometimes when he goes into the foramina that are left by nature (cloacæ) with his probes, he does not find the bone loose, because it may be so held by the granulations that he cannot move it; and yet at the end of three months he chisels down to free it, feeling pretty sure that he will find it detached at that time from the living bone. It may, perhaps, be interesting to allude to modern methods of operation upon such cases in general surgery for a few minutes.

First, I will speak of Neuber's method, or "method of deep canalization." Suppose we have a superficial necrosis of the shaft of the tibia. The granulations all being scraped away and the surface chiselled smoothly and made sterile, a skin-flap is freely dissected up on either side until, without more than very slight tension, these flaps will meet at the bottom, and are fastened to the bone with sterilized nails. If the stretching is not too great, the skin will grow to the subjacent parts, and at the end of a week or ten days the nails can be removed, and the patient is cured. although he has a very deep dimple there; but there is a constant effort on the part of nature to bring that depression up to the surface, and at the end of a year the dimple is a small one, and finally it will disappear altogether.

Secondly, I will allude to the technique of Schede. This is "healing under the moist blood-clot." The cavity is made smooth and absolutely sterile as before. Then a sheet of sterile gutta-percha is placed over the wound, and this is in turn covered with sterile dry dressings, and then a plaster-of-Paris splint encases the limb. Not until then is the rubber tube encircling the thigh removed; whereupon the blood from the small vessels of the bone promptly runs into that cavity and fills it entirely with a solid clot; which last serves as a support, nutriment, and basis for a wonderfully rapid healing process.

Another method is that of Thiersch. Having prepared the cavity thoroughly and destroyed the pus-microbes with peroxide of hydrogen, and got an absolutely smooth surface, we take off skin-grafts, with a razor, from the thigh or elsewhere, covering that bone with such grafts, which will grow to the bone. The bone itself exudes plasma sufficient to nourish them. They generally live as when grafted on normal soft parts, and in about ten days you have that cavity skinned over.

Another plan is that of homogenetic bone-grafting, removing the granulations and perfectly sterilizing, and then filling in the space with the method that Nicholas Senn, among others, advocates,—*i.e.*, with decalcified chips of bone, which do not become revived, but they act as a nutritive material in which connective tissue forms with great rapidity, that connective tissue ultimately forming into bone, so that the cavity will thus commonly heal more quickly than by the old-fashioned method of packing with gauze and waiting for granulations, which takes often months to accomplish, and following which comes slow cicatrization.

Another method is filling with chips of natural bone. Chiselling thin chips lengthwise from the patient's healthy tibia, and then putting them in side by side until the cavity is filled and those are not expected to live, in quite a large percentage of cases being nourished by the vitalized plasma exuding from the raw bone.

The sixth modern method is the heterogenetic method, and probably the most interesting to dentists,—namely, to make use no longer of the idea that the bony cavity will be filled with healthy tissue, but to fill it quickly with something foreign and non-vitalized, like gold fillings in the teeth. Having sterilized the cavity and made undercuts, line it carefully with (for example) gold-foil, and then fill it with plaster of Paris and have it set, and then dissect up a flap on either side and bring the edges together and suture them. That has repeatedly been done, especially in Germany. Of the ultimate results it is too soon to speak positively. There have been more failures thus far than successes, but it is certainly an interesting line of work.

*Dr. Elliott.*—Before we pass the question of necrosis, I would like to say that I have a rather interesting case of a lady who has had a great deal of neuralgic trouble with a left lower molar. I finally advised her to have it removed. She went to one of the specialists and had the tooth taken out. This gentleman told her that she had necrosis of the jaw, and immediately commenced with a bur to cut away the necrosis, the result being that he cut the inferior dental nerve, causing paralysis and a great deal of trouble. Many years ago, in operating on a similar case in London, I noticed this practical difficulty, that in attempting to bur out dead bone the sense of touch was not sufficiently acute to inform me where I left the dead and struck the living. I finally had to wait until the exfoliation occurred.

Dr. Shaw then read a paper entitled "The Preparation of Dental Alloys and Cements."

(For Dr. Shaw's paper, see page 634.)

DISCUSSION.

*Dr. Brockway.*—Do the materials deteriorate by age or exposure to the air?

*Dr. Shaw.*—Yes; the oxide of zinc sometimes changes to carbonate, and I think it would be better to prepare a small portion of liquid at a time.

*Dr. Russell.*—I have tried for many years to make different amalgams. The apparatus Dr. Shaw describes is too elaborate for the ordinary dentist. All text-books teach that the silver should be melted first. A glass furnace is not needed. The tin will carry down the other metals. The lower the heat at which the alloy is melted the more quickly it will set, and it can be melted at the melting-point of tin. I have made an alloy of tin and platinum over a Bunsen burner, so it is hardly necessary to have a plumbago crucible and a glass furnace.

*Dr. O. E. Houghton.*—I attended the meeting of the New York State Dental Society this spring, at Albany, where Dr. Black was the chief attraction with his amalgam experiments. One of the experiments was with tempered amalgam. The experimenter was Dr. Butler, of Buffalo; and Dr. Butler, at the request of Dr. Black, gave his experience before the Society of how it worked, and the results. The amalgam used, I believe, was one of Dr. Black's favorites,—about 68.5 silver, 25.5 tin, 5 gold, and 1 zinc. This amalgam was in an ingot from which he filed off a portion and divided those filings into two parts. Of one portion he immediately made an amalgam filling and inserted it in one of the steel disks which Dr. Black uses for testing. Dr. Butler stated that he had all he could do to get it into the disk before it got hard. He used equal parts in weight of mercury and alloy. That filling showed, under Dr. Black's tests, very little change or contraction forty-eight hours after, and proved to be one of the best fillings tested. Dr. Butler then took the other portion of the alloy, tempered it with boiling water, and mixed it with fifty per cent. in weight of mercury, the same as before; he stated there was a great excess of mercury, and he had to keep squeezing it out for a long time. He found this filling very slow to harden. This filling was tested by Dr. Black forty-eight hours after, and the contraction was found very great, in fact proved one of the worst fillings tested by him. It was spoken of as a very queer thing.

*Dr. Brockway.*—The inference would seem to be that—allowing time to manipulate it—the quicker the amalgam set the better would be the results. If any present have ever used palladium for filling, they will realize that it sets very rapidly,—so quickly that it is difficult to use it in some cases. A considerable quantity of heat is evolved in the operation of setting, so that a mass sometimes explodes; but it makes an admirable filling, so far as the joints are concerned. I never saw an amalgam that made such perfect joints as palladium does, and were it not for the difficulty in manipulating it, and its great cost, I think it would be in general use.

*Dr. Houghton.*—I have had so much experience in making oxychlorides that I am led to differ with Dr. Shaw in relation to the deteriorating of aged zincs. I find that the older it gets the slower it sets, but it sets just as hard. If the powder part is three or four years old, it sets three times as slowly as when freshly made, but that could all be restored to its original condition by recalcining and grinding it again.

*Dr. Howe.*—I feel very glad that Dr. Shaw has presented this subject, for I think it is very desirable that we should know more about the materials we are so constantly using. I am sure there are many dentists throughout the country who would like to make their own alloys, and learn by practical experience the differences in the various formulæ. We are greatly indebted to Professor Black for the information he has given us, resulting from the exhaustive experiments he has carried on; but there is much more for us to know on this subject. Dr. Houghton's remarks in regard to the oxychloride of zinc, recalled to my mind the need that some of us have for slow-setting oxychloride for the filling of root-canals. The filling of these canals with this substance can only be accomplished properly when the cement sets slowly. I would like to ask Dr. Houghton to tell us what, besides aging, will make oxychloride cement set slowly. He has told us that the old powder sets slowly, and how it can be restored to quick-setting; but how can we make that which is quick-setting set slowly?

*Dr. Houghton.*—I must say that has been a matter that has bothered me a great deal. I have succeeded at times and I have failed at other times. It can be done by using various grades of oxide of zinc and manipulation in calcining. I have found in making the best oxychloride that only a percentage of snow-white French zinc should be used. American zinc, which was full of many impurities, was used in making "os artificial" mostly. It contained oxide of iron and various other oxides, and it could not be calcined very long without turning it yellow. The French zinc could be calcined three or four hours and come out perfectly white. I have found that the oxide of iron would produce a beneficial result sometimes in cements in making them set harder. There are as many little freaks in its manufacture as in oxyphosphates, and one must have much experience and tact to have it come out right every time. Hardly two samples of oxyphosphate, even from the same manufacturer, will be exactly alike; but if one gives it his attention, he can get it pretty nearly right each time. Its manufacture is generally left to some assistant, and there is a great deal of carelessness in making it. There is need of a white oxychloride for lining teeth after bleaching and for teeth that are partly discolored, and a slow-setting oxychloride for root-filling. I have thought seriously of bringing out some such preparations, although I have done nothing in that line for nearly twenty years, except for myself and some of my neighbors.

*The President.*—I will call upon Dr. Howell.

*Dr. Howell.*—The subject of splints was mentioned to-night, and I presume many have had the trouble I have of not having a flask deep enough. The ordinary flask is generally much too shallow, and I have designed this one, which I take pleasure in showing, with an extra ring to increase its depth. As will be noticed, it can be either an ordinary flask or one of extra depth. A lady had two broken toes, and the physicians could not regulate them unless they had a rubber splint, which I was called upon to make, this flask proving very useful.

Adjourned.

S. E. Davenport, D.D.S., M.D.S.,
*Editor The New York Institute of Stomatology.*

## Figures and Tables

**Fig. 1. f1:**
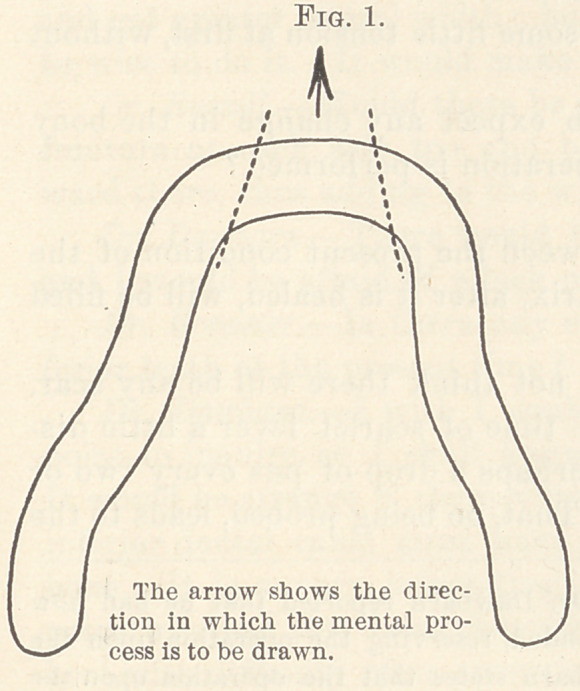


**Fig. 2. f2:**